# Beyond the Iron Throne: exploring the representation of homosexuality in the series *Game of Thrones*

**DOI:** 10.3389/fsoc.2024.1412154

**Published:** 2024-07-18

**Authors:** Gerome Karthic Kumar Louis, Gandhapodi K. Chithra

**Affiliations:** Department of English, School of Social Sciences and Languages, Vellore Institute of Technology, Chennai, India

**Keywords:** representation, homosexuality, identities, thematic analysis, popular media, marginalized groups

## Abstract

**Introduction:**

This research delves into the representation of homosexuality in the television series *Game of Thrones*, adapted from George R.R. Martin's *A Song of Ice and Fire* saga. The study focuses on the navigation and portrayal of homosexual identities and relationships within a historically patriarchal and heteronormative genre.

**Methodology:**

A qualitative research approach, specifically thematic analysis, was utilized to scrutinize the portrayal of homosexuality across all eight seasons and 73 episodes of *Game of Thrones*. The analysis encompassed pivotal scenes, character development, and dialogues related to homosexual representation.

**Findings:**

Thematic analysis revealed several significant themes concerning the representation of homosexuality, including heteronormativity, the interplay of love and war, and the societal and personal impacts of sexual identity. The series portrays the challenges and resilience of homosexual characters, such as Renly Baratheon and Loras Tyrell, against a backdrop of political intrigue and personal ambition. However, the depiction at times veers into sensationalism, which may potentially desensitize viewers to the real-life struggles faced by LGBTQ+ individuals.

**Discussion:**

The study findings suggest that while *Game of Thrones* aims to depict homosexual relationships with complexity and depth, it also risks perpetuating negative stereotypes and sensationalizing trauma for dramatic effect. This dual portrayal underscores the broader challenge within popular media to balance representation with responsible storytelling. The study emphasizes the necessity for more nuanced and empathetic portrayals of marginalized groups to promote greater understanding and inclusivity in media narratives.

## 1 Introduction

The fantasy genre has historically adhered to rigid gender norms and heterosexual storylines. However, *Game of Thrones* diverged from these conventions by incorporating diverse sexual orientations, particularly featuring homosexuality in its narrative. The series offers a complex and diverse storytelling experience with multiple viewpoints and critiques. The show has garnered both acclaim and backlash for its portrayal of women and their dynamics with male characters, as well as the lack of positive representation of LGBTQ+ characters. Nevertheless, it has been lauded for its depiction of multifaceted and resilient female characters who defy traditional gender roles. The Literature Review section provides an overview of current research on gender and sexuality in *Game of Thrones*, highlighting the strengths and weaknesses of existing scholarly discussions. It encompasses a range of studies that examine the representation of femininity, different types of masculinity, and exploration of identities in the series, offering a comprehensive context for the present study's exploration of homosexuality. The method section delineates the qualitative research approach employed in the study, outlining the selection of the series' eight seasons and 73 episodes as the material for analysis. It describes the thematic analysis process used to identify patterns and themes related to homosexual representation. The findings and analysis section is organized into main themes and sub-themes that emerged from the thematic analysis. The primary themes encompass heteronormativity and the intersection of love and war, with sub-themes delving into culturally acceptable forms of masculinity and femininity, sexual identity passing, and the intricate interplay of love, war, and personal ambition within homosexual relationships. The conclusion encapsulates the study's findings and delves into the implications of the representation of homosexuality in *Game of Thrones* for broader societal understandings of sexual diversity in popular media. It reflects on the potential of such representations to challenge heteronormative norms and contribute to more inclusive narratives.

## 2 Review of literature

The portrayal of gender in the series *Game of Thrones* has been examined widely. Sandqvist (2012) aimed to contribute to the literary field by providing an intersectional analysis of the power structures in the series. The focus was on female characters, highlighting the prevalence of male-centric power structures in both societal and familial contexts. Jones (2012) emphasized the importance of using archetypal lenses to analyze the variations in gender norms and societal expectations for women between the novel and the TV series adaptation. Laurie (2015) delved into the controversies surrounding sexual violence in specific episodes of the series, comparing the scenes to their counterparts in the books and exploring the portrayal of rape and its implications in the narrative. Clapton and Shepherd (2017) demonstrated how popular culture can disrupt traditional disciplinary mechanisms and highlighted the gendered nature of political authority. They underscored the reinforcement of gender hierarchies and the gendered foundations of political authority, as exemplified by characters like Daenerys in the series. Ucan (2017) discussed the feminist potential and postfeminist complexity in the representation of women in the story, emphasizing the importance of scholarly works covering various aspects of the series. Singh and Singh (2018) found that the series portrayed subversive and diverse gender and sexual expressions through characters like Lord Varys, Yara, Renly, and Loras. Zare (2019) discovered modest representations of gender role attributes in the characters of *Game of Thrones*, with significant differences between female and male characters, and highlighted the series' challenge of stereotypical gender roles, particularly for female characters.

The representation of women characters in the series has also been a topic of study. Shubham (2022) expressed disapproval of the excessive display of nakedness of women in the show, seeing it as a tactic to retain audience interest. Bollinger-Deters (2018) explored the intersection of high fantasy creative fiction with historical hierarchal oppressions, emphasizing racialized patterns, inclusivity, and the influence of historical contexts on fictional characterizations. Rai (2017) highlighted the contrasting traits and approaches to power between Daenerys and Catelyn, with Daenerys being portrayed as confident and ambitious, relying on external forces, while Catelyn embodies a motherly discourse of power focused on cooperation and empowerment.

Some of the existing literature has contributed to the understanding of the portrayal of masculinity in the series. Johnston (2022) examined how social abjection is used to delegitimize subordinate and marginalized masculinities, suggesting that incorporating abject masculinity (expressions of masculinity that are pushed to the margins of societal norms and become abject) could help challenge patriarchal violence and recover marginalized masculinities. Tan (2018) discussed masculinity as a social construct with various types and representations, including hegemonic masculinity, which is portrayed as the dominant form encouraged by patriarchal society. Characters like Tyrion Lannister represent complicit masculinity, striving for hegemonic traits despite limitations. Ramsay Bolton embodies extreme aggression and sadism, showcasing characteristics of hegemonic masculinity through his brutal and cruel actions (Kehoe, 2021). His crimes and acts of aggression, influenced by notions of hegemonic masculinity, include torture, sexual violence, murder, and psychological manipulation to assert power and dominance. The toxic consequences of hegemonic masculinity^1^ in Westeros society are exemplified through Ramsay's behaviors, indicating a need for a shift away from the glorification of violence and power struggles to prevent further harm and destruction. Evans (2019) highlighted the critique of normatively masculine violence and the exploration of alternative masculine discourses and queer kinships in the fantasy genre. These findings emphasized the potential for challenging patriarchal norms and power dynamics through complex narratives in the series.

The researchers also explored the role of identity to understand the characters better. The role of identity, particularly about gender and sexuality, is a prevalent theme in the field of queer studies and the portrayal of characters in *Game of Thrones*. Identity encompasses how individuals perceive themselves and are perceived by others, shaped by a variety of social, cultural, and personal influences. In the context of this analysis, examining identity is crucial to understanding how characters navigate their sexual orientations and societal expectations within the world of Westeros. Judith Butler's theory of performativity in her book *Gender Trouble* published in 1990 is indispensable in comprehending identity in this context (Butler, 1990). Performativity posits that identity is not an intrinsic essence but rather is shaped through repeated actions and behaviors. This viewpoint is valuable in dissecting characters such as Renly Baratheon and Ser Loras Tyrell, who frequently conceal their homosexual identities to adhere to societal norms of heteronormativity. Their actions mirror Butler's argument that identities are performed and reinforced through social conventions and behaviors. Moreover, the depiction of characters such as Yara Greyjoy in the series challenges conventional gender norms and emphasizes the diversity of gender identity. Yara possesses both typically masculine and feminine characteristics, rejecting rigid gender classifications. This reflects the principles of queer theory, which promotes a broader and more embracing concept of identity (Salih, 2007). Lovric and Hernandez (2019) examined how *Game of Thrones* depicted non-white identities through orientalist and hegemonic lenses, highlighting the perpetuation of power dynamics and stereotypes in the show's character representations. Jones (2009) focused on how queer men experienced their identities, emphasizing the themes of intersectionality, body positivity, political commitments, ideological tensions, and the development of queer consciousness. Olesker (2020) highlighted the importance of identity and normative commitments in determining the success of alliances, noting that the survival of characters within alliances was not guaranteed if their normative commitments did not align with the alliance. The study also suggested that identity and norms were crucial in power transitions and alliance formation.

Kaplan (2017) surveyed the portrayal of sex and non-normative bodies in the television show *Game of Thrones*, with a focus on the character Tyrion Lannister, who had dwarfism. Despite the narrative empowerment and cinematographic adaptations for Tyrion, the show did not treat his sex scenes the same as those of other main characters. *Game of Thrones* demonstrated ambiguity in its approach to pushing boundaries in depicting sex and different body types. Yunara and Kardiansyah (2017) explored the presence of the animus imprint in female characters, specifically Arya Stark and Sansa Stark, from the novel *A Song of Ice and Fire: A Game of Thrones*. The study revealed that both characters exhibit masculine imprints or animus within their characteristics, with Arya Stark displaying the animus personality across the first three stages of animus development.

However, there is a dearth of research that has explored the representation of homosexuality in the series *Game of Thrones* through the prism of various themes. The objective of the thematic analysis was to address the research question: How is Homosexuality Represented within the series *Game of Thrones*?

## 3 Method

To address the research question, the researchers employed qualitative research methodology. Thematic analysis of the series *Game of Thrones* was used to examine important episodes, character development, and dialogues about the representation of homosexuality (Towbin et al., 2004).

### 3.1 Materials

Eight seasons of the series *Game of Thrones* containing 73 episodes in total were selected for the study. The running time for each episode is 50–82 min approximately. The entire show (including the episodes that did not have homosexual content) was analyzed so that the researchers could comprehend the narrative. Viewers can watch episodes in chronological order on streaming platforms such as JioCinema, Max, etc., The streaming service, JioCinema was utilized to access each episode of the series.

### 3.2 Procedure

Thematic analysis was chosen as the primary qualitative data analysis method for identifying patterns across multiple data sources (Alhojailan, 2012; Braun and Clarke, 2012). The procedure of data collection for the thematic analysis in the present study was borrowed from Towbin et al. (2004) in which feature films were analyzed thematically. The show was watched four times in its entirety before it was rewatched exclusively for identifying units of data. A unit of data could be identified from the time frame in which a particular scene took place in an episode of a certain season, relevant to the research question. A unit of data could be extracted from a scene, for example, in which the homosexual lovers Ser Loras Tyrell and Renly Baratheon look at each other and are not able to convey any form of pleasantries or affection toward each other as they do not explicitly acknowledge their homosexual love in front of the crowd leaving them with no verbal input. On the other hand, Ser Loras shows his respect and admiration for Lady Sansa Stark by giving her a flower without hesitation, and the crowd starts cheering for them. It appears that their physical positioning could indicate an attempt to conceal their sexual identity. In addition to visual cues, dialogues among the characters were also recorded as data units. In this case, the speech would be transcribed accurately, and subtitles would be used for greater precision. The collection of visual and verbal data was crucial for capturing implicit messages related to homosexuality.

## 4 Findings and analysis

### 4.1 Representation of homosexuality in *Game of Thrones* (2011–2019)

The characters who exhibited homosexual tendencies were Renly Baratheon, Ser Loras Tyrell, Oberyn Martell, Ellaria Sand, and Yara Greyjoy. Emphasis was given to the characters Renly and Loras for maximum screen space compared to other homosexual characters.

### 4.2 Main theme: heteronormativity

*Game of Thrones* portrays heteronormativity through the actions and beliefs of male and female characters. Heteronormativity refers to the societal expectations that heterosexuality is the standard norm and any deviation from this norm is often stigmatized (Chambers, 2007). The series depicts a negative attitude toward non-heteronormative relationships. The characters Renly Baratheon and Loras Tyrell face discrimination and criticism for their homosexual relationship. This reflects the societal bias against same-sex relationships within the world of Westeros. Here are some of the sub-themes that substantiate the presence of heteronormative concepts in the context of homosexuality.

#### 4.2.1 Sub-theme: the culturally acceptable forms of masculinity and femininity

In *Game of Thrones*, the culturally acceptable forms of masculinity and femininity are deeply embedded in the societal norms and traditions of Westeros. The world of Westeros places a high value on political strength, patriarchy, and physical and sexual dominance over women, associating these qualities with ideal masculinity (Connell, 1995). Firstly, Robert Baratheon's statement from the below extract reflects a hyper-masculine worldview prevalent in the patriarchal society of Westeros:

Robert Baratheon: You ever f^***^ a Riverlands girl? Renly Baratheon: Once. I think. Robert Baratheon: You think? I think you'd remember. Back in our day, you weren't a real man until you'd f^***^ one girl from each of the seven kingdoms and the Riverlands. We used to call it “making the eight” Renly Baratheon: Those were some lucky girls. (S1:E6, units 28–29)

His measure of manhood is tied to sexual conquests, reinforcing traditional gender roles where male prowess is validated through sexual exploits. The notion of “making the eight” implies a crude form of masculinity, where men are expected to assert dominance and control over women. Renly's response, on the other hand, can be interpreted as a mild form of resistance or subversion to his brother's machismo. By acknowledging the girls as “lucky,” Renly subtly challenges the objectification of women inherent in Robert's worldview. However, his response also reflects a level of complicity or conformity to societal norms, as he does not outright condemn Robert's perspective. Furthermore, the emphasis on “making the eight” highlights the importance of regional identity and unity within the Seven Kingdoms. Sexual conquests become a metaphor for political alliances and power dynamics, suggesting that relationships and interactions between individuals are often influenced by broader socio-political contexts. Overall, this dialogue illustrates the complex interplay between gender, power, and identity in the heteronormative world of Westeros:

It could also be inferred that men were not considered real men and they were not conformed to the gender norms of masculinity if they had not had a physical intimation with eight women from different regions. King Robert, who adhered to the concept of heteronormativity in this context, represents “the culturally acceptable form of masculinity.” It appears that Renly provided a false statement after being asked the question about his sexual encounter with a girl from Riverlands. The reason for his lying is not to fall out of gender norms that are considered “normal,” “masculine” and “culturally acceptable” (Courtenay, 2000).

The physical bodies of women are often objectified and used as tools for political alliances or personal gain (Bordo, 1994). For reference, Cersei Lannister could be considered “the culturally acceptable form of femininity” (Schippers, 2007) within the context of the series and one of many who despise homosexual individuals. On several occasions, she expresses her wish of having been born a man:

Cersei Lannister: I should have been born a man. I would rather face a thousand swords than be shut up inside with this flock of frightened hens. Sansa Stark: They are your guests under your protection. You asked them here. Cersei Lannister: It was expected of me as it will be of you when if you ever become Joffrey's queen. (S2:E9, units 27–28)

Cersei Lannister's statement, “I should have been born a man,” reflects a longing for power and agency within the constraints of patriarchal society in Westeros. Cersei's assertion that she should have been born a man suggests that she believes her gender has limited her ability to wield power effectively. In the patriarchal society of Westeros, men traditionally hold positions of authority, while women are often relegated to subordinate roles. Cersei's desire to have been born a man indicates her frustration with these gender-based constraints and her recognition of the privileges afforded to men in her society. Furthermore, Cersei's statement about facing a thousand swords rather than being confined with “frightened hens” underscores her disdain for the perceived weakness of women. She views the other women present as timid and passive, contrasting sharply with her desire for action and agency. This highlights Cersei's rejection of traditional gender norms and her refusal to conform to the expectations placed upon her as a woman.

#### 4.2.2 Sub-theme: sexual identity passing

The concept of sexual identity passing (Harrison, 2013) is merely the concealment of one's gender identity or sexual orientation to pass as what is generally regarded as “normal” in the cultural and sociological contexts. The two most prominent characters who want to fit in the idea of heteronormativity and pass as heterosexuals are Renly Baratheon and Ser Loras Tyrell. The character of Ser Loras Tyrell can be interpreted as a case of sexual identity passing within the context of the story. Despite being openly gay to those close to him, Loras presents himself publicly as the epitome of traditional masculinity, earning him the moniker “The Knight of Flowers.” His outward performance of heterosexual norms and ideals conceals his true sexual orientation from the broader society of Westeros, where such identities are often stigmatized or even punishable:

Sansa Stark: (looking at Ser Loras Tyrell) “The Knight of Flowers” Ser Loras approaches Sansa, sitting on a horse, leaning toward her and giving her a flower. Sansa Stark: Thank you, Ser Loras. Loras looks at Renly. Renly nods at him suggesting that he should go and win the combat. (S1:E5, unit 6)

Sansa Stark's interaction with Loras, coupled with his exchange with Renly, can be viewed through the lens of sexual identity passing. When Sansa refers to Loras as “The Knight of Flowers,” she may not only be acknowledging his renowned chivalry and elegance but also subtly engaging with the facade he presents to the world. By accepting the flower he offers, Sansa participates in the performance of heterosexual courtship rituals, further perpetuating Loras's outwardly heterosexual identity. Loras's glance toward Renly, followed by Renly's nod of encouragement, adds depth to this interpretation. Renly, who shares an intimate relationship with Loras, understands the complexities of Loras's dual identity. His nod can be seen as a silent affirmation of Loras's performance and a reminder to maintain the illusion of heterosexuality, especially in public settings where scrutiny is high. This scene highlights the challenges faced by individuals like Loras who navigate a society that enforces rigid gender and sexual norms. Loras's ability to pass as heterosexual grants him certain privileges and opportunities within the patriarchal power structures of Westeros, but it also necessitates constant vigilance and concealment of his true self. Sansa, Loras, and Renly become a poignant exploration of the complexities of sexual identity passing, the performance of gender roles, and the intersections of power and privilege in the world of *Game of Thrones*.

On the other hand, bisexual characters like Oberyn Martell and Ellaria Sand challenge heteronormativity in contrast to more conservative regions in Westeros. They are also in contrast with what is being called “sexual identity passing.” Oberyn Martell is known as the Red Viper and Ellaria Sand is his paramour from Dorne. They share an open and unconventional relationship and they are both depicted as sexually adventurous and open-minded. Their sexual identity is fluid. The portrayal of Dorne as a region more accepting of diverse sexualities challenges the heteronormative norms seen in the other parts of Westeros.

By featuring characters with diverse sexual orientations, the narrative contributes to the visibility and representation of the LGBTQ+ community to some extent. This representation is crucial in challenging heteronormativity by showing that characters with non-heterosexual orientations can be complex and integral to the story.

### 4.3 Main theme: love and war

As the story progresses, the concepts of love and war become entangled with the characters' struggles with their sexual orientations. The consequences of being perceived as different, whether due to one's sexuality or other factors, are evident throughout the narrative. The themes of love and war (Kumar and Kumar, 2023) are often woven into the fabric of the characters' lives, influencing their decisions and relationships in a world where survival often depends on alliances, loyalty, and the pursuit of power. There are two sub-themes within the main theme Love and War which will be explored below.

#### 4.3.1 Sub-theme: all's fair in love and war

In the following extract, the dynamics between Renly Baratheon and Ser Loras Tyrell can be interpreted through the lens of “All's Fair in Love and War,” highlighting the complexities of power, manipulation, and personal ambition within the context of representing homosexual relationships in the series:

Renly Baratheon: Brienne is a very capable warrior. And she is devoted to me. You are jealous. Ser Loras Tyrell: Jealous? Of Brienne the beauty? Don't make me laugh. Renly Baratheon: (proceeds to undress him) “‘I'll make it up to you.” Ser Loras Tyrell: (gets upset) No, Your Grace. Not tonight. There is another Tyrell who requires your attention. You didn't win my father's support or his army on charm alone. Renly proceeds to have physical intimation with him again. But Ser Loras stops him. Ser Loras Tyrell: Your vassals are starting to snigger behind your back. Brides aren't usually virgins two weeks after their wedding night. Renly Baratheon: And Margaery is a virgin? Ser Loras Tyrell: Officially. Shall I bring her to you? (S2:E3, units 27–29)

Renly's initial attempt to dismiss Loras's concerns about jealousy by seducing him illustrates the blurred lines between love and power in the world of Westeros. Renly, aware of Loras's devotion and loyalty, seeks to placate him through physical intimacy, leveraging their homosexual relationship to maintain control and secure Loras's allegiance. However, Loras's refusal to be swayed by Renly's advances underscores his agency and ambitions within the power dynamics of their relationship. Despite his love for Renly, Loras recognizes the importance of political alliances and the need to prioritize the interests of House Tyrell. His insistence on Renly's attention being diverted to securing the support of other Tyrell family members reflects his understanding of the broader political landscape and the necessity of strategic maneuvering. The exchange between Renly and Loras also reveals the manipulative nature of their relationship, where affection and desire are intertwined with power plays and ulterior motives. Renly's disregard for Loras's concerns about their public perception and his insistence on physical intimacy despite Loras's reservations highlight his sense of entitlement and arrogance as a nobleman. Loras's reference to Margaery Tyrell's virginity and his offer to bring her to Renly further underscores the transactional nature of marriage and alliances in the world of *Game of Thrones*.

In a society where women are often treated as pawns in the game of politics, Loras's willingness to offer his sister's chastity to appease Renly demonstrates the lengths to which individuals are willing to go to advance their own ambitions and secure power. Overall, this scene encapsulates the complex interplay of love, power, and manipulation and the negative representation of homosexuality.

#### 4.3.2 Sub-theme: politics of power

The concept of Politics of Power is significant in understanding homosexuality within the context of the series (Sandqvist, 2012). One cannot function without the other. In this scene from *Game of Thrones*, the dynamics between Margaery Tyrell and Renly Baratheon can be analyzed through “Politics of Power,” illuminating the intricate ways in which individuals manipulate relationships and alliances to consolidate and maintain power. Margaery seeks to use her marriage to Renly as a means of enhancing the power and influence of House Tyrell:

Margaery Tyrell: Your enemies aren't happy about us. They want to tear us apart. And the best way to stop them is to put your baby in my belly. We can try again later. You decide how you want to do it. With me or with me and Loras. However else you like. Whatever you need to do. You are a king. (S2:E3, unit 29)

Margaery's proposition to Renly is a calculated move aimed at solidifying their political union and strengthening Renly's claim to the Iron Throne. By suggesting that Renly impregnate her, Margaery demonstrates her understanding of the importance of heirs in securing dynastic legitimacy and stability. In a world where power is often secured through bloodlines and inheritance, Margaery's offer is a strategic maneuver to bolster Renly's position as a king.

Moreover, Margaery's acknowledgment of their enemies' intentions to undermine their relationship underscores her awareness of the political threats they face. In the ruthless *Game of Thrones*, alliances are constantly tested, and Margaery recognizes the necessity of preemptively addressing any vulnerabilities, here in this case, homosexuality, could be exploited by their adversaries. Margaery's willingness to accommodate Renly's homosexual preferences, whether it be involving Loras Tyrell in their arrangement or allowing Renly to decide the specifics of their intimate encounters, reflects her pragmatic approach to power.

Furthermore, Margaery's reminder to Renly of his status as a king serves as a subtle assertion of her authority and influence within their relationship. Despite Renly's royal title, Margaery asserts her agency and autonomy, positioning herself as an equal partner in their political endeavors rather than a mere pawn or consort.

Overall, this scene exemplifies the “Politics of Power” paradigm by showcasing the strategic calculations, manipulative tactics, and power dynamics at play within the world of *Game of Thrones*. Through Margaery's proposition to Renly, we witness the intersection of personal ambition, dynastic politics, and intimate relationships, highlighting the multifaceted nature of power in the pursuit of the Iron Throne.

There is another instance that exemplifies the politics of power concerning homosexuality in the story. In the following exchange between Tywin Lannister and Olenna Tyrell, the “Politics of Power” reveals the strategic maneuvering and manipulation employed by both characters to advance their respective agendas and maintain control over their family legacies:

Tywin Lannister: The only thing that might turn it are details of your grandson's nocturnal activities. Do you deny that? Olenna Tyrell: Oh, not at all. A sword swallower through and through. Tywin Lannister: And a boy with his affliction should be grateful for the opportunity to marry the most beautiful woman in the kingdoms and remove the stain from his name. (S3:E6, unit 33)

Tywin Lannister's assertion that details of Loras Tyrell's homosexuality illustrate the ruthless pragmatism with which he approaches politics and familial alliances. By leveraging this knowledge, Tywin seeks to coerce Olenna into compliance. Olenna Tyrell's acknowledgment of Loras's homosexuality by saying “a sword swallower through and through” reflects her pragmatic approach to politics and power. As the matriarch of House Tyrell, Olenna understands the importance of securing advantageous alliances for her family, even if it means overlooking or downplaying certain indiscretions or scandals. By openly acknowledging Loras's orientation, Olenna demonstrates her willingness to confront uncomfortable truths and navigate the complexities of courtly intrigue.

Tywin's insistence that Loras should be grateful for the opportunity to marry Cersei and thereby “remove the stain from his name” highlights the transactional nature of marriage alliances in Westeros society. In Tywin's view, the marriage serves as a means to restore Loras's reputation and solidify the alliance between House Lannister and House Tyrell, regardless of the personal feelings or desires of the individuals involved.

Overall, this exchange exemplifies the “Politics of Power” paradigm by showcasing the strategic calculations, manipulation, and negotiation tactics employed by Tywin Lannister and Olenna Tyrell to further their respective interests and maintain control over their family legacies. Through their dialogue, we gain insight into the complexities of power dynamics and alliances within the world of *Game of Thrones*, where personal relationships are often subordinate to larger political ambitions.

### 4.4 Main theme: homophobia

The portrayal of homosexual characters and themes in *Game of Thrones* has been a subject of criticism (Evans, 2019; Hardy, 2019) for its representation and the fate of certain characters. Opinions on this aspect of the show may vary among viewers. Additionally, the probability that anyone could die is worth noting in the story. It is also worth noting that male characters like Renly Baratheon, Oberyn Martell, and Loras Tyrell who have exhibited homosexuality have all been brutally killed in the show in one way or another. It is one of the many aspects which could contribute to the cause of homophobia.

#### 4.4.1 Sub-theme: homosexuals as “degenerates”

In the context of homophobia, there are instances in the early seasons of the show where characters use derogatory language related to homosexuality or exhibit homophobic attitudes. Concepts like homosexuality were not defined precisely in medieval settings (Johansson and Percy, 2009) in the same way as it is in today's contemporary times. The show uses terms relevant to the world it portrays. So, it is important to note that these instances are often portrayed within the context of the medieval society in which the story is set. Words like “homosexuals,” “bisexuals,” “gays,” “lesbians,” “transgenders,” etc. have never existed or been used in the entirety of the series. The most commonly used word for people who exhibit homosexuality is “degenerates,” a negative connotation that could mean “one who has lost or has become deficient in the qualities considered proper to the race or kind; a degenerate specimen; a person of debased” (Oxford English Dictionary, 2024).

In the following exchange, Margaery Tyrell expresses her frustration with her husband, Renly Baratheon, for his apparent disinterest in consummating their marriage and producing an heir. She describes Renly's avoidance of intimacy and his reluctance to engage in sexual activity that could result in conception. This reluctance, according to Margaery, led her to question her fertility and desirability as a wife:

Margaery Tyrell: Whenever I wanted to make a child with him, he had so many excuses, so many late-night war councils. He never wanted to try… except one evening. After he had far too much wine to drink, he suggested something. Something that sounded very painful and couldn't possibly result in children. Maybe the fault was with me. Joffrey Baratheon: No, he was a known…degenerate. Margaery Tyrell: It is such a relief to hear you say so, Your Grace. (S3:E2, units 39–40)

Joffrey Baratheon interjects with the assertion that Renly was a “known degenerate.” Here, the term “degenerate” carries strong connotations of moral decay or deviation from societal norms. Within the framework of homosexuality as degeneracy, Joffrey's statement implies that Renly's homosexuality is inherently immoral or perverse. Margaery's response, “It is such a relief to hear you say so, Your Grace,” can be interpreted as her tacit agreement with Joffrey's characterization of Renly. In a society where heteronormativity is deeply ingrained, Margaery's relief may reflect a sense of reassurance that her marital struggles were not entirely her fault but rather attributable to Renly's perceived deviance.

Language shapes attitudes and beliefs, and using derogatory terms reinforces negative stereotypes and prejudices against homosexual individuals (Carnaghi et al., 2011; Cervone et al., 2021). It perpetuates a culture of intolerance and discrimination, making it more difficult for them to live authentically and without fear of judgment or harm. The term “degenerates” carries strong negative connotations and has been used to demean and dehumanize homosexual individuals like Renly in this context, deemed to be morally corrupt or socially undesirable.

#### 4.4.2 Sub-theme: punishment

In *Game of Thrones*, punishment operates within the framework of a feudal society where justice is enforced by various authorities such as lords, kings, and religious institutions. Punishments can vary widely depending on multiple factors (Harrison, 2013; Evans, 2019), including the nature of the offense, the status of the perpetrator, and the whims of those in power. Common forms of punishment depicted in the series include execution, exile, torture, imprisonment, mutilation, and shaming. It should be noted that being a homosexual is not a predetermined offense with a specific punishment in the series. However, it is considered a perversion by those who exhibit homophobia.

In the story, the Faith Militant also known as Sparrows is a religious order that follows the dominant religion of “The Seven.” The show portrays The Faith Militant as a strict enforcer of their interpretation of the faith's teachings, with a conservative and traditionalist outlook (Benioff et al., 2011–2019). Their stance on homosexuality is one of no tolerance, and they are shown to be expressing this view unequivocally.

The dialogue between the Faith Militant and the man they confront is charged with homophobic slurs and violence, reflecting a society where homosexuality is not only stigmatized but also met with severe punishment:

Faith Militant: C^***^suck^***^! Boy Fuc^***^! You buggering filth! There is a special place in the seventh Hell for your kind. Man: Please. Please. I will pay. I'll pay all of you. Faith Militant: Yes. You will! (slides him with a knife and the man screams). (S5:E4, unit 10)

The Faith Militant's use of derogatory terms like “c^***^suck^***^,” “boy fuc^***^,” and “buggering filth” highlights their deep-seated prejudice against individuals who engage in same-sex relationships or acts. The Faith Militant's verbal abuse serves not only as an expression of disgust but also as a justification for the violence they inflict. By labeling the man as morally corrupt and deserving of punishment, they position themselves as enforcers of societal norms, particularly regarding sexuality. The mention of a “special place in the seventh Hell” further emphasizes the religious condemnation of homosexuality, suggesting that it is not only a social taboo but also a sin deserving of eternal damnation. In this context, the punishment inflicted upon the man for his perceived homosexuality is not only physical but also symbolic of the societal rejection and condemnation faced by LGBTQ+ individuals in such a society. This scene serves as a stark reminder of the discrimination and violence faced by marginalized communities in the pursuit of love and acceptance.

After gathering evidence and testimony, the High Sparrow, the leader of The Faith Militant announces that there is a handful of evidence to proceed with a trial for both Ser Loras Tyrell and Queen Margaery Tyrell. It is important to note that Queen Margaery is one of the few characters in the story who does not view homosexuality as a sin. She has known about her brother's sexuality for a long time. She tries defending her brother in front of The Faith Militant and the High Sparrow by falsely denying him being homosexual, leading to her imprisonment along with her brother:

High Sparrow: The Faith is satisfied there is enough evidence to bring a formal trial for Ser Loras and Queen Margaery. (S5:E6, unit 39)

The show does not delve into why The Faith views homosexuality as a sin. The religion itself has seven aspects, and none explicitly condemns same-sex relationships. As a result, punishments may not always be proportional or just, and innocent characters may suffer as a result of the actions of those in positions of authority.

#### 4.4.3 Sub-theme: trauma

While imprisoned, Loras is subjected to psychological and physical torture, which includes being questioned about his sexual relationships. Loras, faced with the oppressive religious authority of the High Sparrow and the judgmental gaze of the crowd, is compelled to confess to crimes. His confession includes his sexual relationships with other men, particularly Renly Baratheon, as well as his acknowledgment of perjury and various moral transgressions. In this following scene, Loras Tyrell's confession to the High Sparrow can be analyzed through the sub-theme of “trauma”:

High Sparrow: To which crimes will you be confessing? Loras Tyrell: All of them. I lay with other men including the traitor Renly Baratheon (crowd murmuring). I perjured myself before the gods. I am guilty of depravity, dishonesty, profligacy, and arrogance. I see that now. I humble myself before The Seven and accept whatever punishment the gods deem just. (S6:E10, unit 6)

Within the context of the oppressive and heteronormative society depicted in the series, Loras's admission of homosexuality is framed as a grave offense against both religious doctrine and societal norms. The act of confessing to his homosexual acts and other supposed crimes can be seen as a manifestation of the trauma inflicted upon Loras by his society's rejection of his sexual orientation. In this paradigm, the pressure to conform to heteronormative standards and the fear of punishment for deviating from them have caused him profound psychological distress. His willingness to humble himself before the gods and accept punishment reflects not only his internalized shame and self-loathing but also his desperate attempt to seek absolution and alleviate his suffering.

The murmurs from the crowd further underscore the societal stigma attached to homosexuality and the scrutiny to which LGBTQ+ individuals are subjected. Loras's public confession exposes him to ridicule, condemnation, and potentially severe repercussions, amplifying the trauma he experiences as a result of his sexual orientation. The High Sparrow's role in eliciting Loras's confession and determining his punishment highlights the intersection of religious authority and LGBTQ+ oppression. In this paradigm, the religious institution serves as a tool of societal control, perpetuating the trauma experienced by queer individuals like Loras through condemnation and punishment.

It also marks a tragic downfall for his great House Tyrell, as he is the only heir, and without him, his family line will perish. It is evident in this scene that he is traumatized by the High Sparrow and forced to renounce his identity and inheritance:

Loras Tyrell: I will abandon my Tyrell name and all that goes with it. I will renounce my lordship and my claims on Highgarden (crowd murmuring, gasps). I will never marry and I will never father children (crying). (S6:E10, unit 7)

Trauma can have a devastating impact on a person's sense of identity (Nadal and Calvo, 2014; Berman et al., 2020). In Loras's case, he may feel that his identity as a Tyrell is tainted or associated with painful memories, and therefore, he wishes to distance himself from it. This act can be seen as a way to cope with the trauma by disassociating himself from the source of his pain. As a nobleman, Loras likely has responsibilities and expectations tied to his position. Trauma can disrupt one's ability to fulfill these roles and obligations, leading to “feelings of worthlessness and powerlessness” (Lewis, 1992). By renouncing his lordship and claims, Loras may be attempting to relinquish the pressures and burdens that come with his status, seeking a sense of freedom from the expectations placed upon him.

Trauma can also deeply affect one's ability to form and maintain relationships (Goff et al., 2006). Loras fears that his trauma will negatively impact any potential partner or children, or he may feel incapable of providing the love and care they deserve due to his homosexuality. This declaration reflects a sense of hopelessness or resignation regarding his future and his ability to experience intimacy. The emotional intensity of Loras's statement and the reactions of those around him highlight the profound impact of trauma on both the individual and their community (Kaniasty and Norris, 2013). Trauma often evokes strong emotions such as sadness, fear, and sympathy. The reaction of the crowd suggests that Loras's declaration is unexpected and deeply affecting, indicating the significance of his decision in the context of his personal experiences and the wider social context.

Overall, Loras Tyrell's statement encapsulates the complex ways in which trauma can shape an individual's identity, relationships, and future aspirations, in this case, his homosexuality. It reflects a profound sense of loss, despair, and the struggle to find a sense of agency and meaning in the aftermath of traumatic experiences (Ramos and Leal, 2013).

## 5 Discussion

The representation of homosexuality in *Game of Thrones* is a nuanced and complex portrayal within the broader context of gender, power, and societal norms. This analysis synthesizes the primary findings from the study, placing them within the existing literature and emphasizing the implications for LGBTQ+ representation in popular media. *Game of Thrones* operates within a deeply heteronormative framework, where traditional gender roles and heterosexual relationships are the norm. This framework significantly influences the portrayal of homosexual characters and their relationships. The series often intertwines homosexual relationships with political intrigue and power struggles, as exemplified in the relationship between Renly Baratheon and Loras Tyrell. Although their relationship is genuine, it is also instrumentalized within the larger narrative of power dynamics and political alliances, reflecting Clapton and Shepherd's (2017) analysis of gendered political authority in the series, where personal relationships are often subordinated to larger political ambitions. The study reveals that *Game of Thrones* frequently uses derogatory language to refer to homosexual characters, framing them as 'degenerates'. This choice of language, laden with negative connotations, reinforces societal prejudices and homophobic attitudes. The use of such language reflects the medieval setting of the series, where concepts of sexuality are not as nuanced as in contemporary times. Characters like Renly Baratheon and Loras Tyrell are often depicted as morally corrupt or socially undesirable due to their homosexuality, echoing historical attitudes toward non-heteronormative identities (Johansson and Percy, 2009).

The series also delves into the intersection of homosexuality and trauma, particularly through the character of Loras Tyrell. Loras's experiences shed light on how trauma can influence one's identity and relationships. His fears of being unable to express love and care due to his homosexuality underline the profound impact of societal rejection and internalized homophobia. This portrayal resonates with the research of Berman et al. (2020), who examine the interconnectedness of trauma and identity.

While *Game of Thrones* shines a spotlight on the hardships faced by LGBTQ+ individuals, it also runs the risk of sensationalizing trauma for dramatic effect. The graphic and often brutal depiction of homosexual characters could desensitize viewers to the real struggles of these communities. This sensationalism raises concerns about the potential for such representations to diminish the lived experiences of LGBTQ+ individuals, rather than fostering empathy and understanding. This critique is consistent with the broader discourse on the ethical obligations of media in depicting marginalized groups (Sandqvist, 2012; Laurie, 2015).

### 5.1 Implications

The depiction of Renly and Loras' relationship in *Game of Thrones* serves as a poignant reflection of the harsh realities of their world. As their love story becomes intertwined with the political intrigue of the series, it ultimately leads to tragic consequences for both characters. This portrayal serves as a powerful reminder of the challenges and injustices faced by marginalized communities in society, emphasizing the need for greater diversity and inclusivity in popular media. It is important to further examine the broader societal impact of *Game of Thrones*' representation of homosexuality, as the show sheds light on the discrimination and violence faced by LGBTQ+ individuals. However, it is also crucial to acknowledge the potential of the show to sensationalize trauma for shock value, which may desensitize viewers to the real-life struggles of these communities.

### 5.2 Limitations and future directions

The paper acknowledges that there is limited research on queer studies regarding the *Game of Thrones* series, which could indicate a gap in the existing literature. The study mainly focuses on the representation of homosexuality in the show, but it may not cover other aspects or themes of LGBTQ+ representation in depth. Additionally, the paper does not provide a thorough exploration of the historical context of homosexuality in the show or its implications for society, which could offer a more comprehensive understanding of the topic. For future directions, the research could be done by investigating the potential impact of sensationalizing trauma in the show on viewers' perceptions and attitudes toward real-life struggles faced by LGBTQ+ communities, and addressing criticisms and improving their representation in similar media in the future by implementing strategies that promote responsible and inclusive representation.

## Data availability statement

The original contributions presented in the study are included in the article/supplementary material, further inquiries can be directed to the corresponding author.

## Ethics statement

Ethical review and approval was not required for the study on human participants in accordance with the local legislation and institutional requirements. Written informed consent from the patients/participants or patients/participants' legal guardian/next of kin was not required to participate in this study in accordance with the national legislation and the institutional requirements.

## Author contributions

GL: Conceptualization, Data curation, Formal analysis, Investigation, Methodology, Resources, Software, Visualization, Writing – original draft, Writing – review & editing. GC: Funding acquisition, Project administration, Supervision, Validation, Writing – review & editing.

## Footnotes

^1^Abject Masculinity represents a form of masculinity that is “neither subject nor object” as described by Julia Kristeva in Powers of *Horror: An Essay on Abjection* (1982). It is based on the idea of rendering undesirable qualities and entities abject, ostracizing them from acceptable expressions of masculinity and leading society to reject them with horror, disgust, or contempt (Kristeva, 2024).
